# Hypoxia Inducible Factor Signaling Modulates Susceptibility to Mycobacterial Infection via a Nitric Oxide Dependent Mechanism

**DOI:** 10.1371/journal.ppat.1003789

**Published:** 2013-12-19

**Authors:** Philip M. Elks, Sabrina Brizee, Michiel van der Vaart, Sarah R. Walmsley, Fredericus J. van Eeden, Stephen A. Renshaw, Annemarie H. Meijer

**Affiliations:** 1 Institute of Biology, Leiden University, Leiden, The Netherlands; 2 Academic Unit of Respiratory Medicine, Department of Infection and Immunity, University of Sheffield, Western Bank, Sheffield, United Kingdom; 3 Medical Research Council Centre for Developmental and Biomedical Genetics, University of Sheffield, Western Bank, Sheffield, United Kingdom; 4 Department of Biomedical Science, University of Sheffield, Western Bank, Sheffield, United Kingdom; Stanford University, United States of America

## Abstract

Tuberculosis is a current major world-health problem, exacerbated by the causative pathogen, *Mycobacterium tuberculosis* (Mtb), becoming increasingly resistant to conventional antibiotic treatment. Mtb is able to counteract the bactericidal mechanisms of leukocytes to survive intracellularly and develop a niche permissive for proliferation and dissemination. Understanding of the pathogenesis of mycobacterial infections such as tuberculosis (TB) remains limited, especially for early infection and for reactivation of latent infection. Signaling via hypoxia inducible factor α (HIF-α) transcription factors has previously been implicated in leukocyte activation and host defence. We have previously shown that hypoxic signaling via stabilization of Hif-1α prolongs the functionality of leukocytes in the innate immune response to injury. We sought to manipulate Hif-α signaling in a well-established *Mycobacterium marinum* (Mm) zebrafish model of TB to investigate effects on the host's ability to combat mycobacterial infection. Stabilization of host Hif-1α, both pharmacologically and genetically, at early stages of Mm infection was able to reduce the bacterial burden of infected larvae. Increasing Hif-1α signaling enhanced levels of reactive nitrogen species (RNS) in neutrophils prior to infection and was able to reduce larval mycobacterial burden. Conversely, decreasing Hif-2α signaling enhanced RNS levels and reduced bacterial burden, demonstrating that Hif-1α and Hif-2α have opposing effects on host susceptibility to mycobacterial infection. The antimicrobial effect of Hif-1α stabilization, and Hif-2α reduction, were demonstrated to be dependent on inducible nitric oxide synthase (iNOS) signaling at early stages of infection. Our findings indicate that induction of leukocyte iNOS by stabilizing Hif-1α, or reducing Hif-2α, aids the host during early stages of Mm infection. Stabilization of Hif-1α therefore represents a potential target for therapeutic intervention against tuberculosis.

## Author Summary

Tuberculosis is a mycobacterial disease that was a major cause of death until the discovery of antibiotics in the mid-twentieth century. However, TB is once again on the rise, with the emergence of strains that are multi-drug resistant. Mycobacteria are specialists in evading immune cell killing and use host immune cells as a niche in which they can proliferate and survive latently, until subsequent re-activation and spreading causing life-threatening disease. Pharmaceutical reprogramming of the immune system to kill intracellular mycobacteria would represent a therapeutic strategy, effective against currently untreatable strains and less susceptible to drug resistance. Here we use an *in vivo* zebrafish model of TB to show that manipulation of the host genetic pathway responsible for detecting low oxygen levels (hypoxia) causes a decrease in mycobacterial infection. This antimicrobial effect was due to a priming of immune cells with increased levels of nitric oxide, a molecule that is used by immune cells to kill bacteria. Here we show *in vivo* manipulation of a host-signaling pathway aids the host in combatting mycobacteria infection, identifying hypoxic signaling as a potential target for future therapeutics against TB.

## Introduction

Pulmonary tuberculosis (TB), caused by the pathogen *Mycobacterium tuberculosis* (Mtb), is a major world health problem and is a key priority for infectious disease research. The burden of TB has been exacerbated by the increasing occurrence of Mtb strains with resistance to multiple drug treatments, prioritising the need for understanding of the mechanistic basis of host-pathogen interactions during pathogenesis of disease in order to identify novel therapeutic strategies [1]. Upon infection Mtb are rapidly phagocytosed by host leukocytes, but are able to evade bacterial killing mechanisms and utilize the leukocytes as a niche in which to proliferate and disseminate [2]. Leukocyte infection initiates the recruitment of uninfected macrophages, neutrophils and T-cells, to form highly organised structures known as granulomas [3], [4]. Mtb within granulomas can persist for many years and may eventually escape and disseminate during clinical reactivation, causing active disease [5]. The pathogenesis of both initial infection and reactivation of latent infection are not well understood, and further research into host signaling pathways at these stages may uncover novel, host-derived targets for therapeutic intervention against Mtb.

Mycobacterial disease and hypoxia are intimately related. Human tuberculous granulomas are hypoxic environments, and it has been suggested that the relative hypoxia of granulomas contributes to the latent infection phenotype and the associated relative resistance of Mtb to host and pharmacological killing [6], [7]. Hypoxia exerts its effects on host cell signaling predominantly through stabilization of Hypoxia Inducible Factor alpha (HIF-α) transcription factor. HIF-α is stability and activity is regulated by a group of oxygen sensitive enzymes: prolyl hydroxylases (PHDs) and Factor Inhibiting HIF (FIH) [8]–[10]. Oxygen dependent PHD activity leads to degradation of HIF-α, while hypoxia reduces PHD activity, stabilizing HIF-α, which joins a nuclear complex and transduces the hypoxic cellular response [11]. Three HIF-α isoforms have been identified in humans to date, of which HIF-1α is a key regulator of leukocyte function during both inflammation and a range of bacterial infections [12]–[15].

Normal host defense is dependent on HIF-1α expression, which activates and enhances leukocyte functionality [12]. We have previously shown in a zebrafish model of inflammation that stabilized Hif-1α delays inflammation resolution by reducing neutrophil apoptosis and reverse migration at the inflammation site [16]. Existing evidence suggests that the successful clearance of bacterial infections depends on normal HIF-α signaling, and furthermore, immune cell HIF-α is activated by bacterial challenge in normal oxygen levels, demonstrating the fundamental importance of this pathway to immune cell response to invading pathogens [13], [17]. Despite the extensive work done on the effects of hypoxia on Mtb phenotype, the effects of HIF-α stabilization or downregulation in determining the outcome of host-mycobacterial interaction remains unknown, and presents a major research challenge requiring a combination of modern cell biology and genetic approaches in animal models.

The zebrafish is a well-established model organism used to study a wide variety of human diseases [18]. Zebrafish embryos are easily manipulated genetically and their translucency allows for detailed microscopy studies. *Mycobacterium marinum* (Mm), a natural fish pathogen and a close relative of Mtb, causes an infection in zebrafish that mimics key features of human TB, including the formation of caseating granulomas and development of latency [19], [20]. Mm infection of zebrafish embryos has been successfully used to understand both host cell signaling and mycobacterial virulence determinants [21]–[24]. The Hif-α pathway can be manipulated *in vivo* in the zebrafish, both pharmacologically, using non-specific inhibitors of PHD enzymes such as dimethyloxaloylglycine (DMOG), and genetically, by expression of dominant Hif-1α variants [16], [25].

Using the zebrafish Mm model and Hif-α manipulation we aimed to understand the relationship between Hif-α signaling and the outcome of mycobacterial infection. We show that Hif isoforms, Hif-1α and Hif-2α have opposing effects on the host susceptibility to mycobacterial infection by demonstrating that stabilization of Hif-1α and downregulation of Hif-2α signaling decreases bacterial burden of Mm infection. Furthermore, using both genetic and pharmaceutical approaches, we show that this effect acts via a nitric oxide (NO) dependent mechanism. Our findings identify both Hif-α and NO signaling components as potential host-derived targets for therapeutic intervention against TB.

## Results

### Hif-α Signaling Is Detectable at Early Stages of Mm Infection

**Figure 1:**
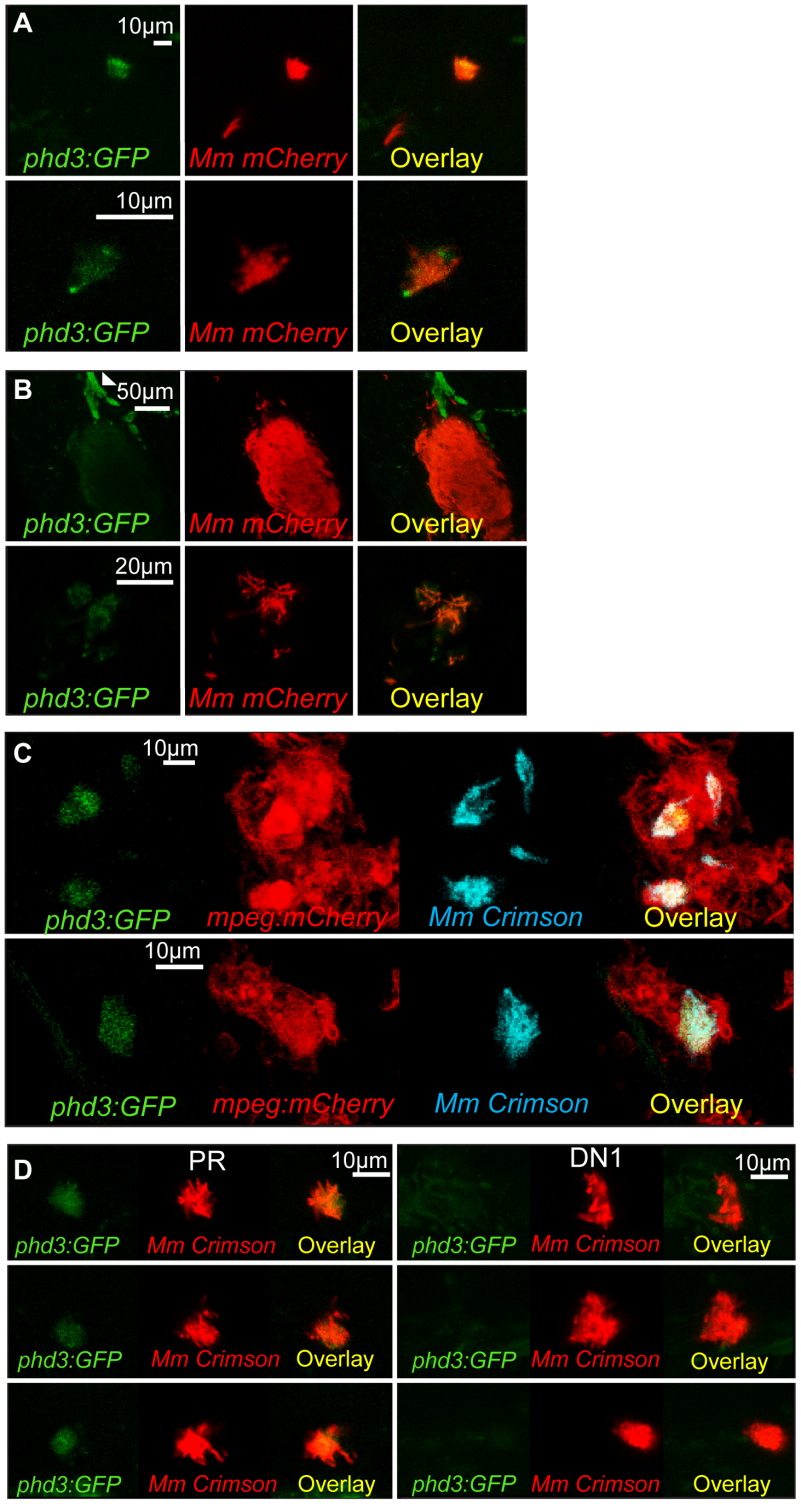
*phd3* is expressed in infected macrophages during early stage Mm pathogenesis in a Hif-1α dependent manner. (A) Fluorescent confocal micrographs of 2 examples of infected areas prior to granuloma formation of 1 dpi embryos (upper and lower panels). *phd3* expression was detected by GFP levels, in green, using the *Tg(phd3:GFP)i144* transgenic line. Mm mCherry is shown in the red channel. Increased levels of *phd3*:GFP expression were detectable in cells associated with infection. (B) Fluorescent confocal micrographs of 2 granulomas at 6 dpi (upper and lower panels). Only low levels of *phd3*:GFP expression were detectable in areas of infection. The low level of GFP is illustrated in the upper panel where the auto-fluorescence of a pigment cell (white arrowhead) is brighter than the phd3:GFP expression. (C) Fluorescent confocal micrographs of 2 embryos with Mm infected macrophages at 1 dpi. The *phd3*:GFP line was outcrossed to the *mpeg1*:mCherry line to show co-localization with infected macrophages. (D) *phd3*:GFP embryos were injected at the 1 cell stage with dominant negative *hif-1αb* RNA (DN1) or phenol red (PR) as a negative control. 60 embryos of each were screened for *phd3*:GFP expression using confocal microscopy and the 3 brightest areas of *phd3*:GFP expression were imaged and showed co-localization with Mm infection. In the DN1 group GFP laser levels and confocal settings were increased until background green fluorescence was visible showing no specific co-localisation with Mm.

HIF-α signaling and its role during mycobacterial infection remain unclear. Upon Mm infection, zebrafish larvae develop early stage granulomas within several days, but levels of Hif-α signaling in early infection and larval granulomas are unknown. To detect levels of Hif-α signaling in Mm infection we utilized in situ hybridization for the known Hif-α target *phd3* (Figure S1A and S1B) and the transgenic line *Tg(phd3:GFP)i144* (Figure 1A and 1B) [16], [25]. At 1 day post infection (dpi) *phd3*:GFP expression was observed in infected leukocytes (Figure 1A). Larval granulomas at 6dpi showed only very low levels of *phd3*:GFP expression and are therefore unlikely to have stabilized Hif-α at this later stage (Figure 1B and S1B). By crossing the *Tg(phd3:GFP)i144* line with a line marking macrophages with a membrane targeted mCherry we showed that the *phd3*:GFP expression was found in infected macrophages at 1 dpi (Figure 1C). This macrophage-specific upregulation of *phd3*:GFP expression by Mm infection was blocked by injection of RNA for dominant negative (DN) *hif-1αb*, indicating this is a Hif-1α dependent host response to Mm infection (Figure 1D).

**Figure 2:**
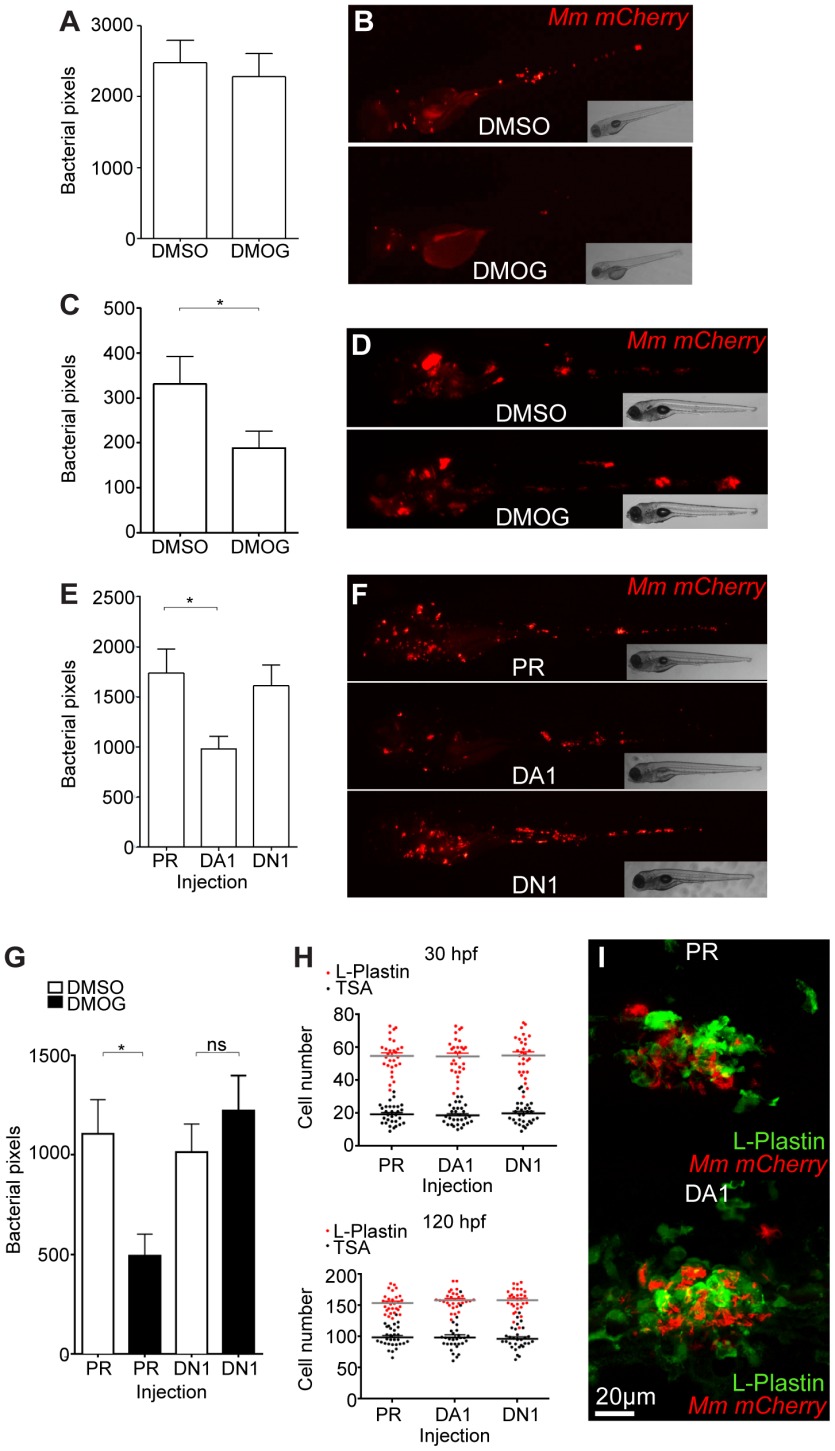
Stabilization of Hif-1α at early stages of infection leads to a decrease in bacterial burden. (A) Quantification of bacterial burden by fluorescent pixel count after DMOG treatment between 5 and 6 dpi. No significant difference was observed between groups. Data shown are mean ± SEM, n = 106–121 accumulated from 3 independent experiments. (B) Stereo-fluorescence micrographs of Mm mCherry infected 6 dpi larvae from (C). (C) Bacterial pixel counts of larvae treated with DMOG between -4 and 24 hpi, imaged at 4 dpi. DMOG treated embryos have significantly lower levels of bacterial burden. Data shown are mean ± SEM, n = 109–114 accumulated from 3 independent experiments. *P<.05, **P<.01, and ***P<.001. (D) Fluorescence micrographs of representative infected larvae for data shown in (E). (E) Bacterial pixel counts of RNA injected larvae at 4 dpi. Dominant active *hif-1αb* (DA1) significantly decreased bacterial burden in infected larvae compared to both phenol red injected controls (PR) and dominant negative *hif-1αb* (DN1). Data shown are mean ± SEM, n = 115–127 as accumulated from 3 independent experiments. (F) Example fluorescence micrographs from the data found in (E). (G) Bacterial pixel counts of RNA injected larvae at 4 dpi after DMSO/DMOG treatment for 24 hours at 4 hours before Mm injection. Dominant negative *hif-1αb* was able to block the antimicrobial effect of DMOG treatment. Data shown are mean ± SEM, n = 83–100 as accumulated from 3 independent experiments. (H, upper panel) L-plastin (macrophages and neutrophils) and TSA (neutrophils only) wholebody counts at 30 hpf. No significant difference was observed between groups. Data shown are mean ± SEM, n = 90 accumulated from 3 independent experiments. (H, lower panel) L-plastin and TSA wholebody counts at 120 hpf. Data shown are mean ± SEM, n = 90 as accumulated from 3 independent experiments. (I) Example fluorescence confocal micrographs of 4 dpi granuloma structures from phenol red (PR) and dominant active *hif-1αb* (DA1) injected larvae. Leukocytes are identified by Alexa-488 (green) labeled L-plastin antibody.

Stabilization of Hif-α by DMOG treatment between 5 and 6 dpi did not reduce the bacterial burden at 6 dpi (Figure 2A and 2B), suggesting that it is specifically early Hif-α stabilization which may represent a novel cellular defense mechanism to arm the leukocytes and cause Mm killing.

### Stabilization of Hif-1α Signaling at Early Stages of Infection Decreases Mm Burden

Using a combination of pharmacological and genetic approaches to upregulate Hif-1α signaling we aimed to determine the effects on pathogenesis in the zebrafish Mm model. Treatment with DMOG from 4 hours pre-infection to 24 hours post infection significantly decreased bacterial burden compared to DMSO negative control embryos, assessed at 4 dpi by fluorescent imaging and pixel count analysis (Figure 2C and 2D). This is consistent with stabilized Hif-1α aiding the host to combat Mm infection. To eliminate the possibility that DMOG affects bacterial growth we performed an *in vitro* assay for 24 hours of treatment, which showed that DMOG does not affect bacterial growth in culture, assessed by both OD^600^ reading and by plating to check viability (Figure S2A and S2B).

Hif-1α signaling was manipulated genetically by injecting RNA for dominant active (DA) and dominant negative (DN) *hif-1αb* variants [16], [26], [27]. Due to a genome duplication event in zebrafish there are two homologues of human *HIF-1α*, *hif-1αa* (ZFIN: *hif1αa*, NCBI Reference Sequence: XM_001337574.2) and *hif-1αb* (ZFIN: *hif1αb*, NCBI Reference Sequence: NM_200233.1) [28]. *hif-1αb* aligns more closely to the human sequence than *hif-1αa*, and contains all three highly conserved hydroxyl-regulated amino acids, while *hif-1αa* only has two. We have previously reported that dominant *hif-1αb* constructs are able to modulate Hif-1α signaling and that *hif-1αb* is the homologue responsible for the delay in resolution of inflammation observed when Hif-1α signaling is stabilized [16], [25]. Therefore all dominant constructs used in this study are based on the *hif-1αb* homologue. Injection of DA *hif-1αb* RNA into one-cell stage embryos, followed by injection of Mm at 1 day post fertilization (dpf), caused a decrease in bacterial burden at 4 dpi, when compared to both phenol red (PR) injected controls and DN *hif-1αb* injected embryos (Figure 2E and 2F). The reduction in bacterial burden was comparable to that seen in DMOG treatment embryos (Figure 2C and 2D). Furthermore, injection of DN *hif-1αb* blocked the anti-mycobacterial effect of DMOG without affecting the dynamics of infection in untreated wild-type fish (Figure 2G). Leukocyte numbers were unaffected by dominant *hif-1αb* constructs at the timepoints of Mm injection (30 hpf) and imaging (120 hpf, Figure 2H and 2I). These data indicate that Hif-1α stabilization aids the host in clearing Mm infection independently of a change in leukocyte number. Although bacterial burden was decreased by stabilized Hif-1α, general granuloma structure was morphologically normal in DA *hif-1αb* injected larvae compared to controls (Figure 2I). The observation that granulomas are able to form in the presence of stabilized Hif-1 α suggests that in these fish, changes in pathogenesis occur before the appearance of larval granulomas. These data show that overexpression of stabilized Hif-1α pre-infection and during initial infection reduces the host's susceptibility to Mm, therefore we focused our further investigations on early stage Mm pathogenesis.

### Nitrosylation Levels Are Increased in Unchallenged Leukocytes with Stabilized Hif-1α

Once phagocytosed by leukocytes, pathogens are subjected to a number of bactericidal processes, including exposure to reactive nitrogen species (RNS) [29]. Due to the highly reactive nature of RNS, direct measurement of production has previously proved problematic. To overcome this an anti-nitrotyrosine antibody has been used in other fish models [30], and we have previously shown that this antibody can be used in wholemount zebrafish embryos after infection [31]. The advantage of measuring nitrosylation of tyrosine is that this is a stable protein formation, therefore detecting historical NO (nitric oxide) production.

**Figure 3:**
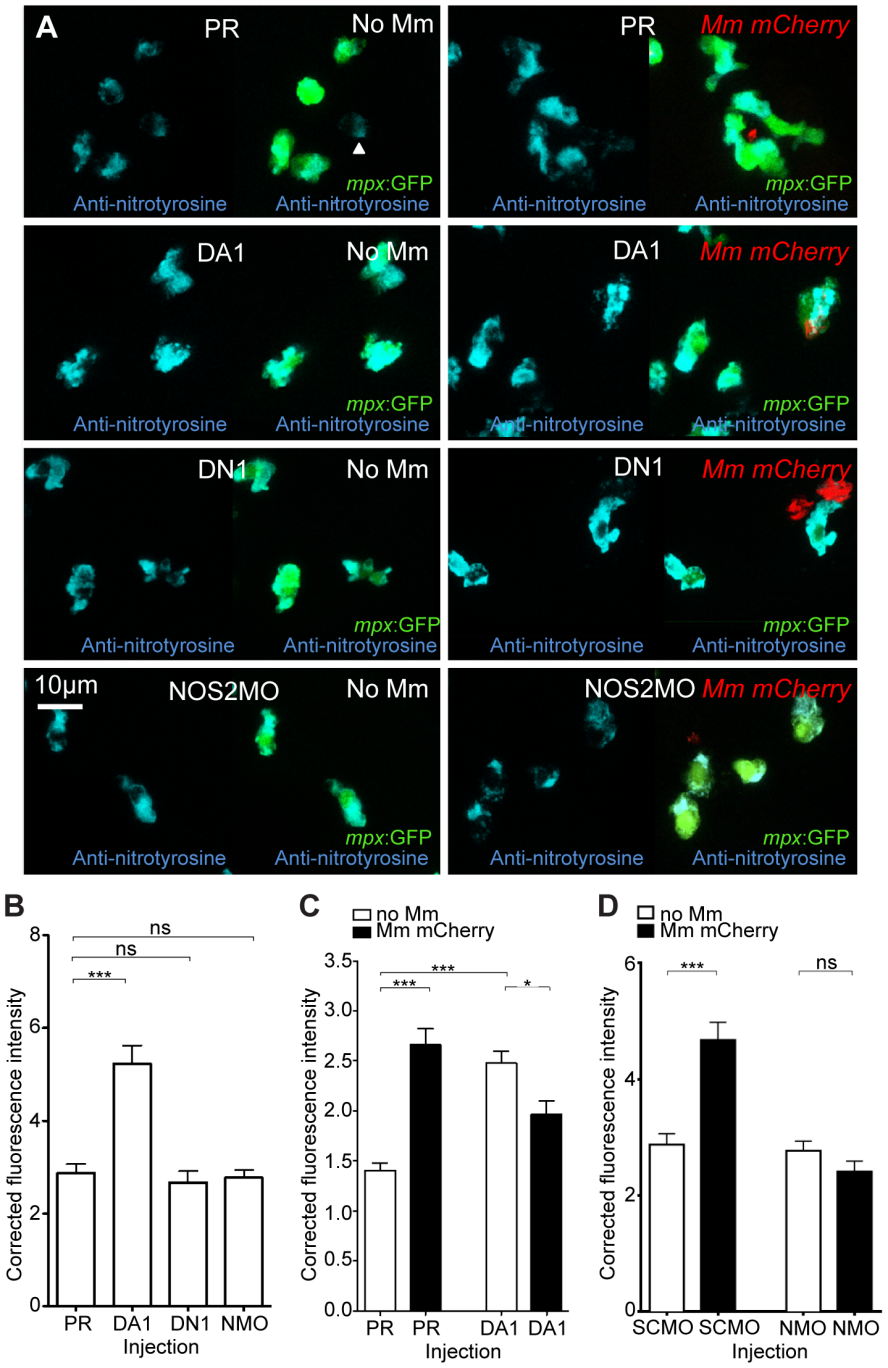
Nitrosylation levels are increased in unchallenged leukocytes with stabilized Hif-1α. (A) Example fluorescence confocal z-stacks of the caudal vein region of embryos stained with Alexa-633 labeled anti-nitrotyrosine antibody (blue), imaged at 1 dpi in the presence or absence of Mm infection. Embryos were injected with phenol red (PR), dominant active *hif-1αb* (DA1), dominant negative *hif-1αb* (DN1), or *nos2a* morpholino (NOS2MO). Anti-nitrotyrosine mainly co-localized with neutrophils (labeled with *mpx*:GFP), however, some cells without GFP (white arrowhead), possibly macrophages, were also labeled. (B) Corrected fluorescence intensity levels of anti-nitrotyrosine antibody confocal z-stacks in uninfected larvae at 2 dpf (1 dpi equivalent). Dominant active *hif-1αb* (DA1) had significantly increased anti-nitrotyrosine levels in the absence of Mm bacterial challenge compared to phenol red (PR) injected controls. Data shown are mean ± SEM, n = 67–92 cells accumulated from 5 embryos per group. Graph shown is a representative dataset of 3 independent experiments. (C) Corrected fluorescence intensity levels of anti-nitrotyrosine antibody confocal z-stacks of dominant active *hif-1αb* (DA1), or phenol red (PR) control injected embryos in the presence or absence of Mm infection at 1 dpi. Data shown are mean ± SEM, n = 233–270 cells accumulated from 15 embryos. Graph shown is combined data from 3×5 embryos from independent experiments. (D) Corrected fluorescence intensity levels of anti-nitrotyrosine antibody confocal z-stacks of *nos2a* morpholino (NMO) or standard control morpholino (SCMO) injected embryos imaged at 1 dpi (2 dpf) in the presence or absence of Mm. Data shown are mean ± SEM, n = 46–92 cells accumulated from 5 embryos. Graph shown is a representative dataset of 3 independent experiments.

In the absence of infection, we found that anti-nitrotyrosine staining was specific to leukocytes at 2 dpf. As with other fish species, staining was found mainly in neutrophils, but also in macrophages (Figure 3A). Nitrosylation levels are higher in neutrophils, partly due to a contribution of neutrophil-specific myeloperoxidase, which is able to nitrosylate tyrosine residues in the absence of peroxynitrite [30]. In the absence of infection, nitrosylation levels at 2 dpf were significantly higher after DA *hif-1αb* injection, compared to control and DN *hif-1αb* injected embryos (Figure 3A and 3B), suggesting that DA *hif-1*α leukocytes are already in an activated state prior to infection. In control embryos, the presence of Mm infection at 1 dpi (the equivalent timepoint of 2 dpf) increased nitrosylation levels in leukocytes (Figure 3A and 3C). Nitrosylation levels of DA *hif-1αb* leukocytes remain high in infected embryos compared to uninfected controls, but are lower than DA *hif-1αb* uninfected equivalents (Figure 3A and 3C).

### Neutrophil-Specific Expression of Stabilized Hif-1α Causes Increased Neutrophil Nitrosylation

**Figure 4:**
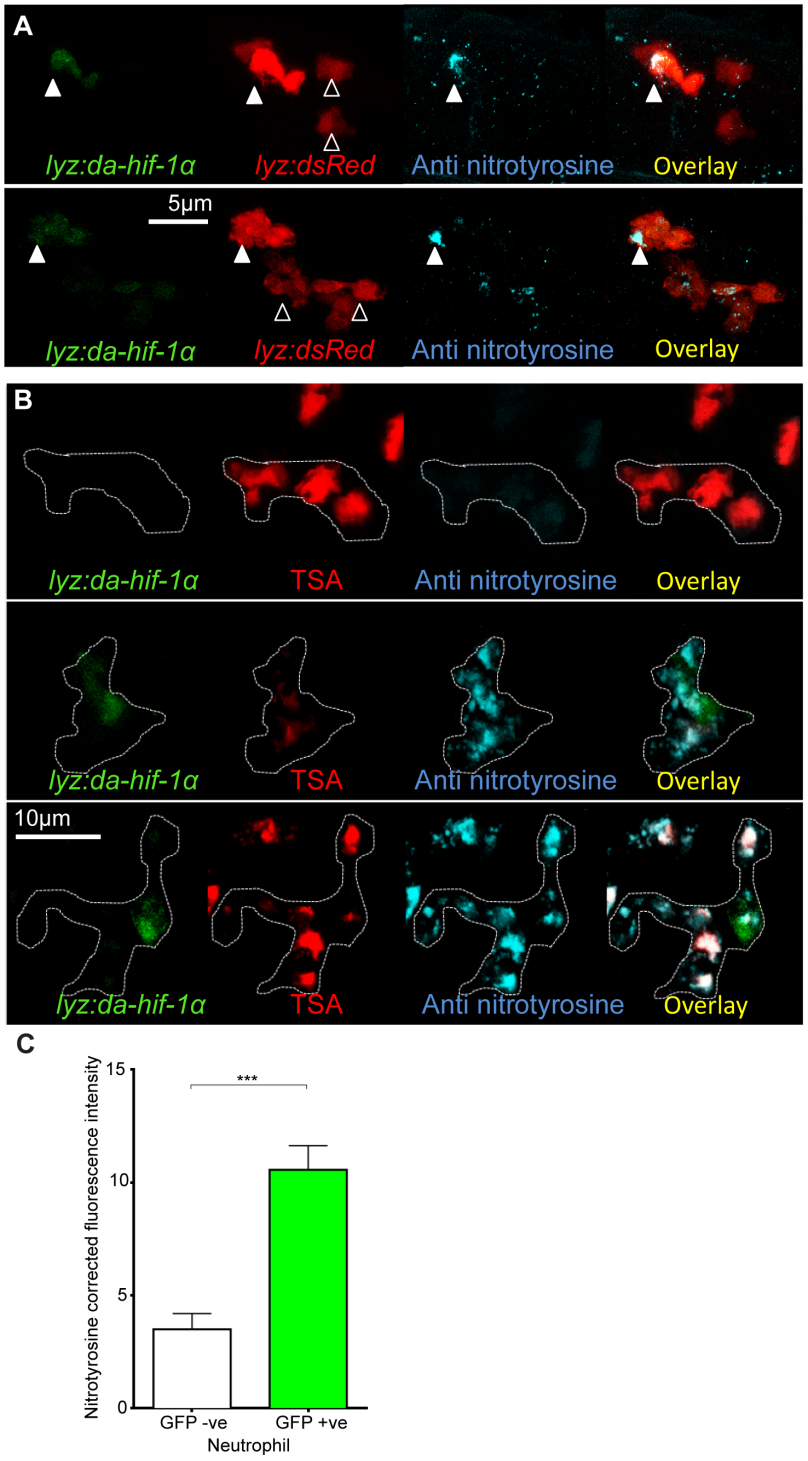
Neutrophil-specific stabilization of Hif-1α causes increased neutrophil nitrosylation. (A) Confocal photomicrographs of expression of *lyz* driven DA *hif-1αb* and *IRES- nlseGFP* (*lyz:da-hif-1α*) in lyz:dsRed embryos stained with anti-nitrotyrosine antibody at 2 dpf. Cells with nuclear localized eGFP colocalized with lyz:dsRed expression showing neutrophil specificity of the transgenic construct. Mosaic labeled neutrophils (white arrowheads) had a higher level of anti nitrotyrosine signal compared to GFP negative ones (black arrowheads). (B) Confocal photomicrographs of a *Tg(lyz:da-hif-1αb:IRES-nlsegfp)* (*lyz:da-hif-1α*) injected ABTL embryo at 2 dpf. TSA staining of endogenous myeloperoxidase was used to stain neutrophils. Due to the nature of the myeloperoxidase staining, the whole cell is not marked, so the cell boundaries have been traced (dotted line) using brightfield z-stacks. The upper panel shows an example of a *lyz:da-hif-1α* negative neutrophil with low levels of anti-nitrotyrosine. The lower panels show two examples of *lyz:da-hif-1α* positive neutrophils in the same embryo, exhibiting a greater level of anti-nitrotyrosine compared to the negative neutrophils. (C) Corrected fluorescence intensity levels of anti-nitrotyrosine antibody confocal z-stacks of GFP negative or positive neutrophils in embryos transiently expressing *Tg(lyz:da-hif-1αb:IRES-nlsegfp)*. Embryos were imaged at 2 dpf. For each GFP positive neutrophil observed, a neighboring GFP negative neutrophil was also imaged. Data shown are mean ± SEM, n = 20 cells per group accumulated from 13 embryos from 4 independent experiments. P values were calculated using a paired T-test.

Systemic stabilization of Hif-1α by DA *hif-1αb* RNA injection increased nitrosylation levels in neutrophils (Figure 3A). In order to test the cell autonomy of the increase in nitrosylation observed after stabilization of Hif-1α, we transiently injected DNA constructs using neutrophil and macrophage specific promoters to express DA *hif-1αb* in mosaic. To express DA *hif-1αb* in specific leukocyte lineages a lysozyme C (*lyz*) promoter was used to drive expression in neutrophils (*Tg(lyz:da-hif-1αb,IRES-nlsGFP)* construct, subsequently referred to as *lyz:da-hif-1αb*), and an *mpeg1* promoter was used to drive expression in macrophages (*Tg(mpeg1:da-hif-1αb,IRES-nlsGFP)* construct, subsequently referred to as *mpeg:da-hif-1αb*) [32], [33]. Both constructs contained a nuclear-localized GFP to visualize the mosaic expression in neutrophils or macrophages, expressed separately from the *hif-1αb* via an internal ribosome entry site (IRES). When these constructs were injected into neutrophil or macrophage reporter lines expected leukocyte-specific, mosaic expression was observed (Figure 4A, S3A, and S3B).

When *lyz:da-hif-1αb* DNA was injected into *Tg(lyz:DsRED2)nz50* embryos or wildtype embryos (with neutrophils labeled with a TSA post-mortem stain), GFP positive neutrophils had higher levels of nitrotyrosine compared to GFP negative neutrophils in the vicinity (Figure 4A–C). These data support a link between stabilized Hif-1α and increased tyrosine nitrosylation in neutrophils. Although mosaic expressed *mpeg:da-hif-1αb* localized specifically to macrophages (Figure S3B), GFP expression did not correlate to an increase in anti-nitrotyrosine. We previously observed that neutrophils represent the majority of cells with anti-nitrotyrosine labeling, although some *mpx*:GFP negative cells were labeled (Figure 3A, white arrowhead). We confirmed that there are a proportion of macrophages labeled with anti-nitrotyrosine in the wildtype infected and uninfected scenario, although the frequency of labeling was much lower than observed for neutrophils (Figure S3C).

These data indicate that neutrophils are the main cell type that have tyrosine nitrosylation, and that this nitrosylation can be increased by stabilizing Hif-1α specifically in neutrophils.

### Decrease in Mm Burden Caused by Stabilized Hif-1α Is Dependent on iNOS

RNS are produced by the activity of the nitric oxide synthase (NOS) enzymes. There are three characterized forms of NOS, namely endothelial-NOS (eNOS), neural-NOS (nNOS), and the leukocyte specific inducible-NOS (iNOS). iNOS expression has been shown to be increased in infected leukocytes, and is present in zebrafish leukocytes [34].

**Figure 5:**
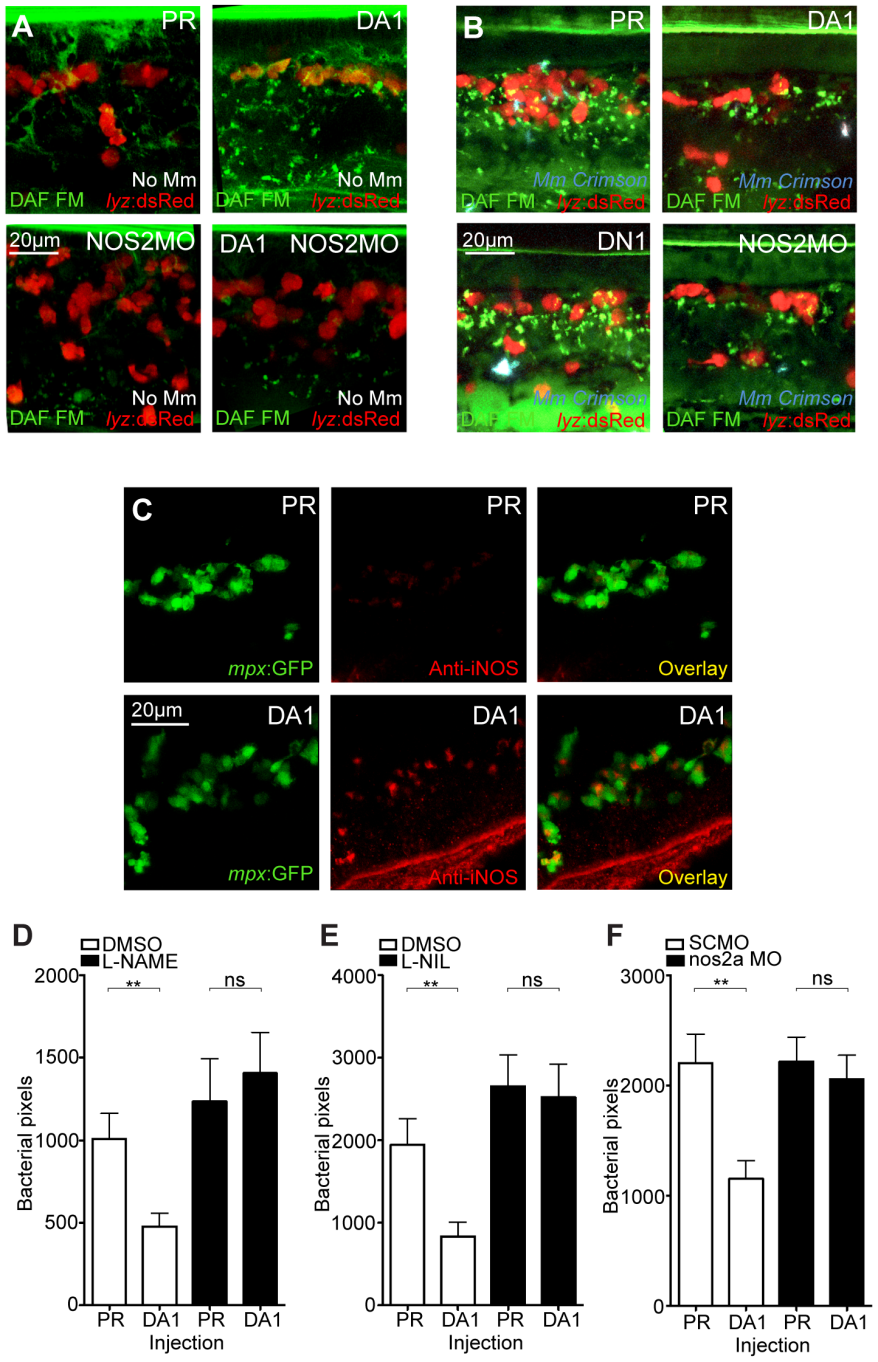
Hif-1α mediated anti-mycobacterial effect is iNOS dependent. (A) Fluorescent confocal micrographs of DAF-FM DA stained embryos at 2 dpf in the absence of infection. Neutrophils are identified by *lyz*:dsRed expression. DAF-FM DA staining between the timepoint of infection and 1 dpi produced varying levels of background and stained cells of the central nervous system (neurons and notochord) as well as having leukocyte-associated staining. The line of staining at the top of each image is DAF-FM DA staining in the notochord. Punctae of DAF-FM DA show upregulation of NO signaling Dominant active *hif-1αb* (DA1) embryos had more punctae than phenol red (PR) controls. *nos2a* morpholino (NOS2MO) reduced the punctae in both the PR and DA1 background. (B) Fluorescent confocal micrographs of DAF-FM DA stained embryos at 2 dpf in the presence of Mm infection. In phenol red (PR) controls DAF-FM DA punctae are increased. DAF-FM DA staining is not specific for iNOS (it is a pan-NOS probe), and the *nos2a* morpholino (NOS2MO) was not able to downregulate all of the DAF-FM DA staining after Mm infection, although punctae number were reduced. The number of punctae was also reduced in the dominant active *hif-1αb* (DA1) injected embryos after Mm infection. Dominant negative *hif-1αb* (DN1) caused no change in punctae in the presence of Mm infection compared to PR controls. (C) Fluorescent confocal micrographs of iNOS antibody staining in mpx:GFP embryos. Phenol red (PR) injected controls had very low levels of anti-iNOS antibody staining. Dominant active *hif-1αb* (DA1) had increased levels of anti-iNOS antibody, a stain which was mainly neutrophil specific. (D) Bacterial burden at 4 dpi after injection of DA *hif-1αb* (DA1) or phenol red control (PR) and treatment with the pan-NOS inhibitor L-NAME. Data shown are mean ± SEM, n = 62–89 as accumulated from 3 independent experiments. (E) Bacterial burden at 4 dpi after injection of DA *hif-1αb* (DA1) and treatment with the iNOS inhibitor L-NIL. Data shown are mean ± SEM, n = 60–87 as accumulated from 3 independent experiments. (F) Bacterial burden at 4 dpi after co-injection of DA *hif-1αb* and the *nos2a* morpholino, using the standard control (SCMO) morpholino as a negative control. Data shown are mean ± SEM, n = 109–116 as accumulated from 4 independent experiments.

Increase of nitrosylation levels in neutrophils after infection is likely to be due to increased iNOS as the morpholino against *nos2a*, the zebrafish gene for iNOS [34], was able to abrogate the increase in nitrotyrosine levels following infection (Figure 3D). To confirm that the increase in nitrosylation observed after DA *hif-1αb* is due to iNOS we used a biochemical probe for NO and an antibody stain for iNOS. DAF-FM DA is a probe that measures NO levels directly [35]. In the absence of Mm, DAF-FM DA staining was increased after RNA injection of DA *hif-1αb* compared to controls (Figure 5A). The signal of DAF-FM DA staining increased after Mm infection, indicating iNOS activity (Figure 5B). Both the increasing effect on DAF-FM DA of DA *hif-1αb* in the absence of infection, and the increase after infection in controls, could be partially blocked using the *nos2a* morpholino, illustrating the iNOS specificity of these effects (Figure 5A and 5B). Furthermore, using an iNOS antibody [36], we were able to detect higher levels of iNOS protein in the DA *hif-αb* neutrophils in non-infected embryos, which were present only at low levels in controls (Figure 5C).

To assess the effect of blocking iNOS activity at the 1 dpi stage on bacterial burden at 4 dpi, we treated early infected embryos with NOS inhibitors. NOS activity was inhibited using the pan-NOS inhibitor L-N^G^-Nitroarginine methyl ester (L-NAME) and the iNOS specific inhibitor N6-(1-iminoethyl)-L-lysine (L-NIL) at early stages of Mm infection [34], [37], [38]. Bacterial burden at 4 dpi was not significantly affected by either treatment in control embryos, although there was a trend towards increased infection levels after NOS inhibition (Figure 5D and 5E). Both inhibitors were able to block the decreasing bacterial burden effect of DA *hif-1αb* at 4 dpi (Figure 5D and 5E). Morpholino knockdown of *nos2a* was also able to block the decreased bacterial burden in DA *hif-1αb* injected embryos compared to PR injected controls (Figure 5F). These data confirm that the positive effect of stabilized Hif-1α on the host to combat Mm infection is dependent on iNOS.

### Hif-1α and Hif-2α Have Opposing Effects on Mm Burden in an iNOS Dependent Mechanism

In humans there are three different HIF-α transcription factors: HIF-1α, HIF-2α, and HIF-3α. It is becoming clear that HIF-2α is important in leukocyte biology [39], [40]. In order to investigate the effects of *hif-2*α modulation, we synthesized dominant variants for *hif-2*α with the equivalent hydroxylation site mutations to the *hif-1*α variants [16]. As with Hif-1α, Hif-2α has two homologues in the zebrafish, *hif-2αa* (ZFIN: *epas1a*, NCBI Reference Sequence: XM_690170.5) and *hif-2αb* (ZFIN: *epas1b*, GenBank:DQ375242). Unlike *hif-1α* homologues, *hif-2α* sequences are highly similar and both contain all three conserved hydroxylation sites. DA *hif-2αa* increased *phd3* expression by in situ hybridization and *phd3*:GFP expression at 1 dpf, whilst DN *hif-2*α*a* decreased expression levels (Figure S4A,B). DN *hif-2αa* also blocked the increase in *phd3* expression associated with early Mm infection at the 1 dpi stage of pathogenesis (Figure S4C). These data illustrate that zebrafish Hif-2α has similar effects on a well characterized target of the Hif-α transcription factor, *phd3*, as the previously characterized zebrafish Hif-1α [16], [25].

**Figure 6:**
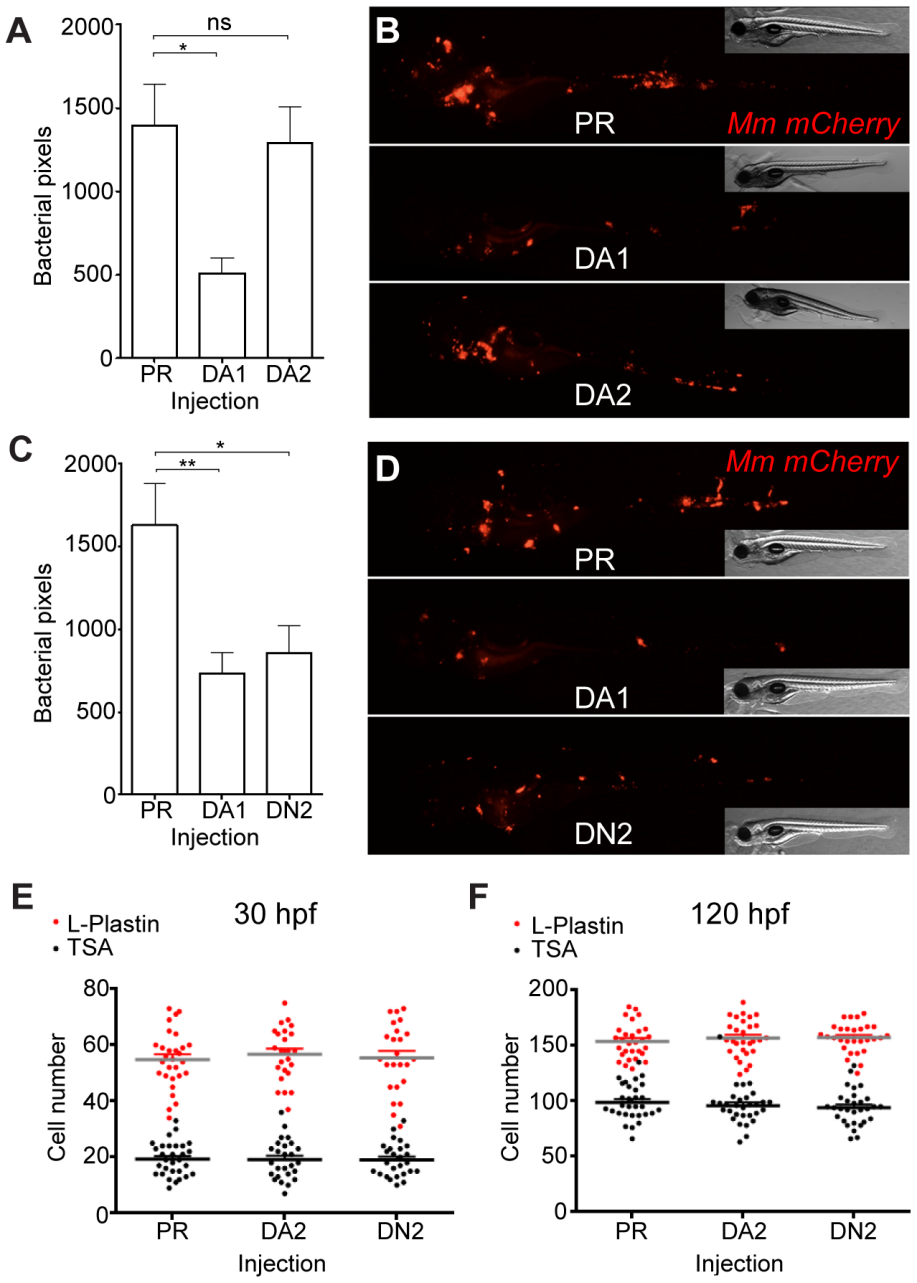
Hif-2α has opposing effects on bacterial burden than Hif-1α. (A) Bacterial pixel counts of dominant active *hif-2αa* (DA2) bacterial burden levels in 4 dpi infected embryos compared to phenol red (PR) and dominant active *hif-1αb* (DA1) injected controls. Data shown are mean ± SEM, n = 52–79 as accumulated from 3 independent experiments. (B) Example fluorescence micrographs of the data shown in (A). (C) Bacterial pixel counts of dominant negative *hif-2αa* (DN2) bacterial burden levels in 4 dpi infected embryos compared to phenol red (PR) and dominant active *hif-1αb* (DA1) injected controls. Data shown are mean ± SEM, n = 74–82 performed as 3 independent experiments. (D) Example fluorescence micrographs of the data shown in (C). (E) L-plastin (macrophages and neutrophils) and TSA (neutrophils only) wholebody counts at 30 hpf after injection of dominant active (DA2) and dominant negative (DN2) *hif-2αa* RNA. No significant difference was observed between groups. Data shown are mean ± SEM, n = 77–82 as accumulated from 3 independent experiments. (F) L-plastin and TSA wholebody counts at 120 hpf after injection of dominant active (DA2) and dominant negative (DN2) *hif-2αa* RNA. Data shown are mean ± SEM, n = 87–90 as accumulated from 3 independent experiments.

**Figure 7:**
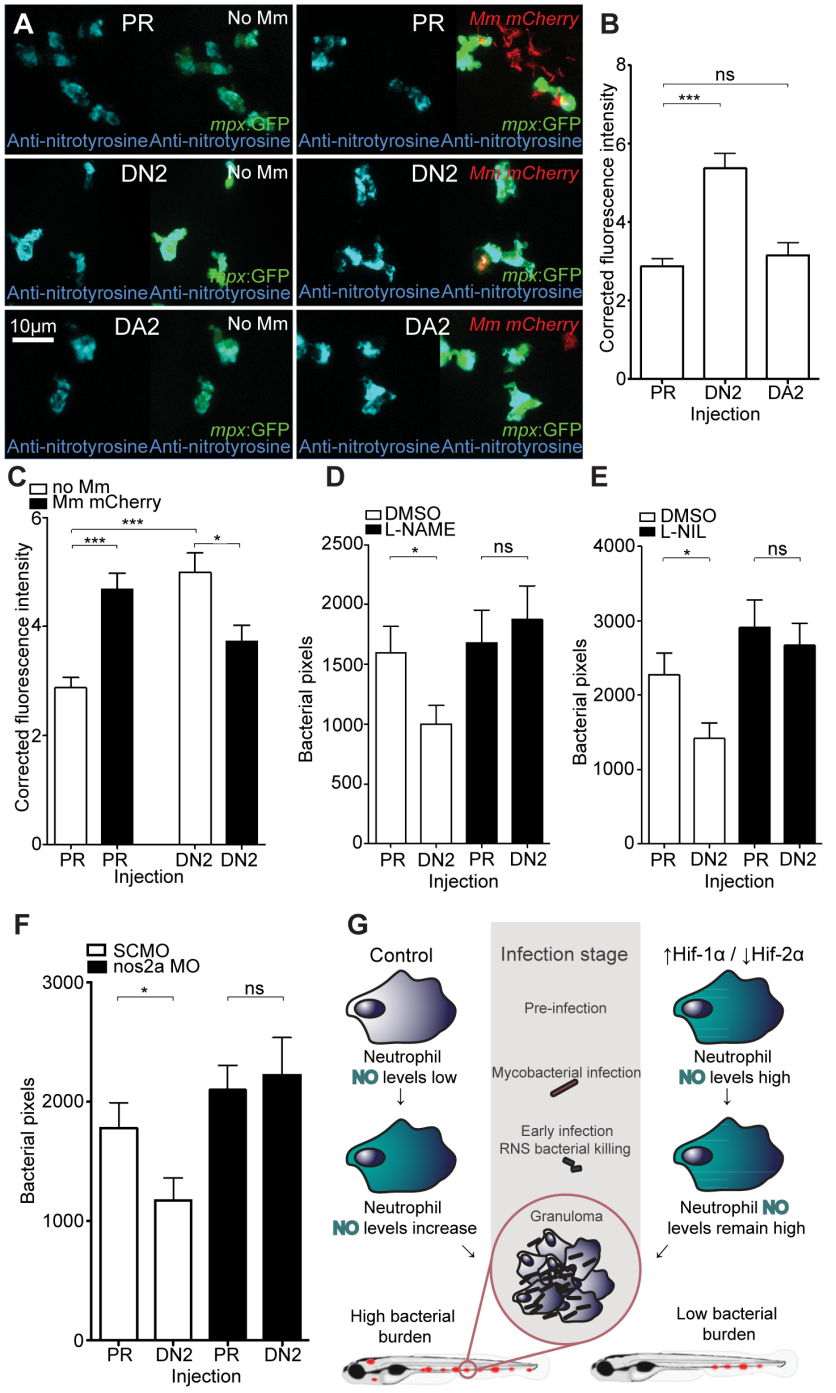
Dominant negative Hif-2α decreases bacterial burden via an iNOS dependent mechanism. (A) Example fluorescence confocal z-stacks of the caudal vein region of embryos stained with anti-nitrotyrosine antibody, imaged at 1 dpi (2 dpf), in the absence and presence of Mm infection. Embryos were injected with dominant negative *hif-2αa* (DN2), dominant active *hif-2αa* (DA2), or phenol red (PR). (B) Corrected fluorescence intensity levels of anti-nitrotyrosine antibody confocal z-stacks in uninfected larvae. Dominant negative *hif-2αa* (DN2) had significantly increased anti-nitrotyrosine levels in the absence of Mm bacterial challenge compared to phenol red (PR) injected controls. Data shown are mean ± SEM, n = 42–92 cells accumulated from 5 embryos. Graph shown is a representative dataset of 3 independent experiments. (C) Corrected fluorescence intensity levels of anti-nitrotyrosine antibody confocal z-stacks of dominant active *hif-2αa* (DA2), or phenol red (PR) control injected embryos in the presence or absence of Mm infection at 1 dpi (2 dpf). Data shown are mean ± SEM, n = 46–92 cells accumulated from 5 embryos. Graph shown is a representative dataset of 3 independent experiments. (D) Bacterial burden at 4 dpi after injection of dominant negative *hif-2αa* (DN2) or phenol red control (PR) and treatment with the pan-NOS inhibitor L-NAME. Data shown are mean ± SEM, n = 67–85 as accumulated from 3 independent experiments. (E) Bacterial burden at 4 dpi after injection of dominant negative *hif-2αa* (DN2) and treatment with the iNOS inhibitor L-NIL. Data shown are mean ± SEM, n = 52–58 as accumulated from 3 independent experiments. (F) Bacterial burden at 4 dpi after co-injection of dominant negative *hif-2αa* (DN2) and the *nos2a* morpholino, using the standard control (SC) morpholino as a negative control. Data shown are mean ± SEM, n = 103–109 as accumulated from 4 independent experiments. (G) Schematic of the effect of early Hif-α modulation during early Mm infection.

DA *hif-2αa*, while able to increase *phd3* expression, had no effect on bacterial burden compared to controls (Figure 6A and Figure 6B). DN *hif-2αa* reduced bacterial burden at 4 dpi to a similar level to that of DA *hif-1αb* (Figure 6C and 6D). As was the case with dominant *hif-1αb* constructs, dominant *hif-2αa* variants had no effect on leukocyte numbers (Figure 6E and 6F). Nitrosylation levels in DN *hif-2αa* were found to be high in non-infected leukocytes, as with DA *hif-1αb*, with no effect of DA *hif-2αa* (Figure 7A and 7B). Mm infection caused a decrease in nitrosylation levels in DN *hif-2αa* leukocyte, as with DA *hif-1αb* (Figure 7C). The reduction in bacterial burden seen with DN *hif-2αa* could be blocked by inhibition of iNOS (Figure 7D–7F) indicating that the reciprocal effects of Hif-1α and Hif-2α modulate susceptibility to mycobacterial infection via an iNOS dependent mechanism (Figure 7G).

## Discussion

The rise in prevalence of multi-drug resistant TB creates an urgent need for novel, host-targeting therapies to complement existing antibiotics and to combat currently untreatable strains of Mtb [41], [42]. Using a well-established zebrafish/Mm infection model of TB, we have identified a new anti-mycobacterial leukocyte phenotype being driven by Hif-1α stabilization and consequent iNOS activity. Our data identify that *in vivo* manipulation of Hif-α, a ubiquitous host-signaling pathway, can affect specific cellular mechanisms of pathogen handling, tipping the host-pathogen balance in favour of the host to decrease mycobacterial infection.

Mtb infection and Hif-α signaling have only previously been linked in the context of the hypoxic, necrotic center of the fully formed caseating granuloma. The necrotic center has been previously identified in adult zebrafish granulomas, however larval granulomas are not necrotic, and to our knowledge the levels of Hif-α signaling in larval granulomas had not previously been investigated [19]. We did not detect high levels of Hif-α signaling, via elevated *phd3* target gene expression, in larval granulomas. However, upregulated expression of *phd3* was observed in infected macrophages at early stages of pathogenesis, before the formation of larval granulomas, and we were able to demonstrate that this increase in expression is Hif-α dependent. Upregulation and stabilization of HIF-1α is a known consequence of leukocyte activation during onset of infection, even in normoxia [13], and remains high during pathogenesis of other types of bacterial infections leading to enhanced leukocyte function [13], [15]. In contrast, we observed levels of Hif-α signaling in early Mm infection, but not at the granuloma stage, suggestive of a silencing of Hif-α signaling over the course of Mm pathogenesis. This observation is consistent with leukocyte transcriptional reprograming observed in human mycobacterial disease and with murine macrophages having upregulated HIF-1α in the presence of heat-killed Mtb but not in the presence of viable Mtb [43]–[45]. A dampening effect on Hif-α signaling could be a mechanism by which mycobacteria are able to form a protected niche in which to proliferate and disseminate, and this may be via manipulation of transcription factors such as HIF-1α. This possibility is supported by dominant negative *hif-1αb* having no effect on Mm bacterial burden, suggesting that Hif-1α signaling does not play a major role in leukocyte anti-mycobacterial activity during the normal pathogenesis of infection. Stabilization of HIF-1α during the pathogenesis of mycobacterial infection may represent a therapeutic opportunity to re-arm leukocytes and to inhibit further infection.

The study of the zebrafish/Mm larval granuloma model has led to key discoveries for TB pathogenesis causing changes in treatment practises in the clinic [42], [46]. However, the zebrafish embryo is an untapped resource for study of mycobacterial pathogenesis at the earliest stages of infection, before granuloma structures form. Genetic manipulation of Hif-1α during mycobacterial infection has not previously been explored, and further understanding of the roles of this critical host-signaling pathway may uncover Hif and its signaling components as future therapeutic targets for intervention against TB. A critical role of HIF-1α during bacterial infection was demonstrated in murine knockout studies showing that HIF-1α signaling is required for proper response to *Streptococcal* bacterial challenge [47]. Furthermore localized treatment with a drug that stabilized HIF-1α led to a decrease in proliferation of the skin pathogens, *Pseudomonas aeruginosa* and *Acinetobacter baumanii*, in a mouse abscess model [48]. Our data demonstrate, *in vivo*, that pharmacological or genetic stabilization of Hif-1α can aid the host in the fight against mycobacterial infection. Importantly, these data suggest that therapeutic upregulation of HIF-1α signaling could complement current antibiotic treatments in the fight against Mtb infection.

In the zebrafish Mm model, the early treatment window of DMOG (treatment between -4 hpi and 24 hpi, followed by wash-off then bacterial burden assessment at 4 dpi) and the DA *hif-1αb* RNA injection at the one-cell stage, (an effect that will be diluted as the embryo develops), indicate that Hif-1α stabilization at early stages of Mm infection, pre-granuloma formation, is causing the decrease bacterial burden at 4 dpi. This is further supported by the observation that embryo granulomas are able to form after DA *hif-1αb* injection despite a decrease in bacterial burden. Therefore, to understand the mechanism of action of early Hif-1α stabilization, we focused on the role of bacterial killing by the leukocytes during early stage infection. A major mechanism of bacterial killing is the use of RNS by leukocytes [29], [49]. The leukocyte enzymatic producer of NO, iNOS, is a tightly regulated enzyme, and is a known target of a number of immune transcription factors, including HIF-1α [40]. We found that levels of NO, as assessed by protein nitrosylation, were consistently higher in neutrophils of infected embryos at early stages of infection. Morpholino reduction of iNOS confirmed that increased nitrosylation in neutrophils after infection was iNOS dependent. We showed that stabilization of Hif-1α is able to activate neutrophils to produce NO in the absence of bacterial challenge and that this could be achieved through neutrophil specific expression of DA *hif-1αb*, demonstrating a cell-autonomous effect. The increased level of NO in non-infected embryos with stabilized Hif-1α indicates a priming of neutrophils to bacterial challenge, leading to greater levels of RNS pre-infection. Early bacterial killing would lead to lower levels of bacterial survival, decreased dissemination and the observed decrease in bacterial burden at the granuloma stages of infection. This early priming of neutrophils could be blocked using iNOS inhibition, confirming that the reduced susceptibility to Mm infection due to Hif-1α stabilization is dependent on iNOS activity.

The role of neutrophils in early mycobacterial infection is not fully understood. Macrophages have previously been thought the major leukocyte involved in the pathogenesis of mycobacterial infection [50] and are also the main leukocyte-type involved in the phagocytosis of intravenously injected mycobacteria and are present in abundance in the granuloma. However, more recently, potentially important roles of neutrophils during mycobacterial infection are becoming evident. Neutrophils are known to be able to undertake oxidative killing of Mm phagocytosed by macrophages in embryonic granulomas, a mechanism which may be important to control infection, however their role at early stages of infection has not been addressed [51], [52]. We observed that enhanced nitrotyrosine levels early after infection were mostly detected in neutrophils, although in rare instances nitrosylation levels were detectable in both uninfected and infected macrophages. This was confirmed using the iNOS antibody, which also showed a mainly neutrophil localization. In the absence of infection there are detectable levels of nitrotyrosine in neutrophils, likely due to myeloperoxidase activity, an enzyme that is neutrophil-specific in zebrafish [53]. Myeloperoxidase can form NO-derived inflammatory oxidants and it has been shown that myeloperoxidase is responsible for the majority of tissue nitrosylation in fish in the absence of infection [30], [54].

The anti-microbial effect of stabilized Hif-1α was abrogated after pharmacological and morpholino inhibition of iNOS. Neutrophils form the major leukocyte population that display protein nitrosylation after stabilization of Hif-1α, therefore we hypothesize that through an unknown mechanism, increased neutrophil iNOS levels leads to increased Mm killing at early stages of infection, ultimately decreasing bacterial burden. As discussed above, macrophages are the major leukocyte involved in the phagocytosis of Mm in this model [50]. However we found many instances where neutrophils contain internalized Mm in the first 24 hours post infection. Our observations indicate that neutrophils are able to internalize Mm before the presence of granuloma structures, and that they have elevated nitrosylation levels during infection. Therefore, a potential mechanism is that neutrophils are able to phagocytose Mm and increase iNOS after Hif-1α stabilization leading to enough early bactericidal activity to significantly reduce bacterial burden at later timepoints. However, it is clear that in the zebrafish embryo model, as in other models, macrophages are the major cell type with internalized Mm at early timepoints of infection. Therefore, a more likely hypothesis is that neutrophils with activated iNOS are able to interact with infected macrophages, either by transfer of live bacteria or transfer of reactive nitrogen species, leading to increased bactericidal activity. We therefore hypothesize that neutrophils play an important role alongside macrophages in early Mm bacterial killing, but the bactericidal mechanisms of this interaction are yet to be uncovered.

The role of Hif-2α isoform stabilization in leukocytes during inflammation and infection has not been widely investigated in any *in vivo* model of infection. We set out to identify what effect Hif-2α modulation had on the outcome of Mm infection. Although stabilization of Hif-2α has the same increasing effect as Hif-1α stabilization on expression of the major Hif-α target gene, *phd3*, we observed an opposing effect on Mm bacterial burden. We demonstrate that the decrease in bacterial burden was due to an increase in neutrophil nitrosylation after downregulation of Hif-2α. The decrease in bacterial burden after Hif-2α downregulation could be blocked by early iNOS inhibition and we hypothesize that this effect is mediated by differential roles of the Hif-α isoforms on the iNOS genetic pathway. HIF-1α and HIF-2α have been previously been shown to have opposing effects on iNOS in mammalian cultured macrophages, where HIF-1α stabilization transcriptionally upregulated iNOS, while HIF-2α stabilization decreased NO levels [40]. Our findings confirm that Hif-2α is able to upregulate the well-characterised Hif-α target *phd3* while having opposing effects on nitrosylation, corroborating previous *in vitro* observations [40]. These data demonstrate the *in vivo* consequence on bacterial infection of the differential regulation of iNOS by Hif-α variants. The opposing effects of Hif-α isoforms on bacterial burden highlight the tight control of NO homeostasis in leukocytes. The potential of the HIF-α pathway for therapeutic intervention in other diseases, including cancer and ischemia, is widely recognized, however differential roles of HIF-α isoforms in these diseases are only recently coming to light [55], [56]. These regulatory mechanisms, in part mediated by HIF-α, are complex and further studies are required before the regulation of HIF-α isoforms and iNOS during infection is fully understood.

In conclusion, our data demonstrate that *in vivo* modulation of host Hif-α signaling during early Mm pathogenesis can lead to decreased burden of mycobacterial infection. Stabilization of Hif-1α, or reduction of Hif-2α, results in priming of neutrophil NO bactericidal activity leading to lower mycobacterial burden after challenge with infection. Our data highlight the delicate balance of HIF-α and iNOS signaling in leukocyte function during infection and highlight the important role of neutrophils during early stage Mm infection. Further understanding of the complex crosstalk between Hif-α and iNOS pathways during Mtb infection will help identify novel, host-targeted, therapeutic strategies against TB. NO priming of neutrophils by targeted Hif-α modulation, may decrease the level of initial Mtb infection and act to block the development of acute TB disease caused by re-activation and dissemination of latent Mtb infection. Host targeted strategies would be predicted to be beneficial against all types of TB, including multiple drug resistant strains, and may be less susceptible to therapy-resistance than antibiotic strategies, thereby reducing the global burden of TB.

## Materials and Methods

### Ethics Statement

Zebrafish lines were handled in compliance with the local animal welfare regulations and maintained according to standard protocols (zfin.org). The breeding of adult fish was approved by the local animal welfare committee (DEC) of the University of Leiden. All protocols adhered to the international guidelines specified by the EU Animal Protection Directive 2010/63/EU.

### Zebrafish and Bacterial Strains

Zebrafish were maintained according to standard protocols [57] and local animal welfare regulations. Strains used were ABTL (wildtype), *Tg*(*phd3:GFP*)*i144*, *Tg(mpx:GFP)i114*, *Tg(lyz:DsRED2)nz50* and *Tg(mpeg1:mCherryF)ump2* [25], [32], [58].

Infection experiments were performed using *M. marinum* strain M (ATCC #BAA-535), containing the pSMT3-mCherry or pSMT3-Crimson vector [59]. Liquid cultures were prepared from bacterial plates [59]. Injection inoculum was prepared in 2% polyvinylpyrrolidone40 (PVP40) solution (CalBiochem) as previously described [60], [61]. 100 colony-forming units (CFU) of bacteria were injected into the caudal vein at 28 hpf as previously described [61].

### Whole Mount *in situ* Hybridization of *phd3*

Whole mount *in situ* hybridization of *phd3* was carried out as previously described [16], [25].

### Confocal Microscopy of Tg(*phd3:GFP*)^i144^ Larvae

6 dpi and 1 dpi Tg(*phd3:GFP*)^i144^ [25] larvae infected with Mm were embedded in 1% low melting point agarose (Sigma Aldrich) and transferred to a Leica DMIRBE inverted microscope with a Leica SP1 confocal scanhead for imaging with 40 or 63 times lenses.

### Pharmacological Stabilization of Hif-α with DMOG

The pan hydroxylase inhibitor, DMOG (dimethyloxaloylglycine, Enzo Life Sciences), was used at a 100 µM concentration as previously described [16]. DMSO solvent controls were used. Unless otherwise stated embryos were treated from 4 hours pre Mm infection to 24 hpi by addition to the embryo water. The inhibitors were then washed off with fresh embryo water and grown to 4 dpi for assessment of bacterial load as described below.

### RNA Injections of Hif-α Variants

Embryos were injected with dominant Hif-α RNA at the one cell stage as previously described [16]. *hif-α* variants used were dominant active (DA) and dominant negative (DN) *hif-1αb* (ZFIN: *hif1ab*) and *hif-2αa* (ZFIN: *epas1a*) (primer sequences in Table S1). Phenol red (Sigma Aldrich) was used as a vehicle control.

### Stereoimaging and Bacterial Pixel Count

Embryos were imaged at 4 dpi on a Leica MZ16FA Fluorescence Stereo Microscope. Brightfield and fluorescence images were generated with a Leica DC500 (DFC420C) camera. Bacterial loads were analysed using dedicated pixel counting software as previously described [23].

### Leukocyte Staining

Larvae were fixed in 4% paraformaldehyde in PBS overnight at 4°C and leukocytes were immune-labeled using the l-plastin antibody as previously described [60], [62]. Neutrophils were labeled with TSA (TSAplus kit, Fluorescence Systems, Perkin Elmer Inc) staining labeled neutrophils in fluorescein green fluorescence as previously described [63]. Two timepoints were chosen for this analysis, the timepoint of Mm injection (28–30 hpf) and the timepoint of bacterial burden assessment (5 dpf). RNA groups were blinded prior to counting. Neutrophils and leukocytes in embryos and larvae were counted in the tail region using a Leica MZ16FA Fluorescence Stereo Microscope.

### Morpholino Knockdown of *nos2a*

The *nos2a* morpholino (Genetools) was used as previously reported [34]. A standard control morpholino (Genetools) was used as a negative control.

### Antibody Staining to Detect NO Markers

Larvae were fixed in 4% paraformaldehyde in PBS overnight at 4°C and nitrotyrosine levels were immune-labeled with a rabbit polyclonal anti-nitrotyrosine antibody (Merck Millipore 06-284) at a 1∶200 dilution of primary antibody, and were detected using an Alexa Fluor (Invitrogen Life Technologies) secondary antibody.

Larvae were fixed in 4% paraformaldehyde in PBS overnight at 4°C and iNOS was immune-labeled with a rabbit polyclonal iNOS antibody (BD Biosciences) as previously described [36]. Detection was with goat anti-rabbit HRP-conjugated antibody (Abcam, 1∶500 dilution) and Cy3Plus TSA kit (Perkin-Elmer).

### Confocal Microscopy and Quantification of Corrected Cell Fluorescence of Anti-Nitrotyrosine Levels

Embryos were imaged at 1 dpi, in the presence or absence of infection, embedded in 1% low melting point agarose (Sigma Aldrich) and transferred to a Leica DMIRBE inverted microscope with a Leica SP1 confocal scanhead for imaging with 40 or 63 times lenses. For quantification purposes acquisition settings and area of imaging (in the caudal vein region) were kept the same across groups. Corrected total cell fluorescence was calculated for each immune-stained cell using Image J as previously described [64]. The GFP of the *Tg(mpx:GFP)i114* [58] was used to assess the leukocyte cell boundaries.

### Mosaic Expression of DA *hif-1αb* in Neutrophils and Macrophages

The Tol2kit multisite gateway-based transposon system was used to make transgenic constructs to transiently and mosaic express DA *hif-1α* specifically in neutrophils and macrophages [65]. DA *hif-1αb* was recombined into the middle entry pDONR221 using BP Clonase (Invitrogen). An LR Clonase (Invitrogen) Gateway reaction was performed with p5E-*lyz* (neutrophil specific promoter) or p5E-*mpeg1* (macrophage specific promoter, [33]), pDONR221-*da-hif-1αb* and p3E-IRES-nlsEGFPpA inserted into pDestTol2pA2. The resulting plasmids, *Tg(lyz:da-hif-1αb,IRES-nlsGFP)* and *Tg(mpeg1:da-hif-1αb,IRES-nlsGFP)*, were microinjected with tol2 transposase RNA into the one cell stage embryo to express in transient, mosaically. Positive fish were screened for the transgene using the heart marker eGFP expression (found in the Gateway vector), and positive cells were screened by confocal microscopy for the nuclear localized eGFP signal showing the expression of the transgene.

### DAF-FM DA Staining

Embryos were injected with the relevant *hif-α* construct and infected with Mm at 1 dpf. At the timepoint of infection DAF-FM DA was applied to the embryo water as previously described [35]. DAF-FM DA was washed off using embryo water at 1 dpi and imaged using confocal microscopy.

### Pharmacological Inhibition of NOS

The pan-NOS inhibitor L-NAME, (NG-Nitro-L-arginine methyl ester, Tocris Bioscience), was used at 200 µM as previously described [34], [37]. The iNOS inhibitor L-NIL (N6-(1-iminoethyl)-L-lysine, dihydrochloride, Tocris Bioscience) was used at a 200 µM concentration [38]. DMSO solvent controls were used at corresponding concentrations for each treatment. Unless otherwise stated embryos were treated from 4 hours pre Mm infection to 24 hpi by addition to the embryo water. The inhibitors were then washed off with fresh embryo water and grown to 4 dpi for assessment of bacterial load as described above.

### Dominant *hif-2αa* Cloning

Dominant active zebrafish *hif-2αa* was generated by successive rounds of site directed mutagenesis, each mutating a hydroxylation site into a non-hydroxylatable form as previously described for *hif-1αb* [16]. Dominant negative *hif-2αa* was generated by a truncation at the equivalent amino acid to the 330^th^ amino acid in the human sequence, as previously described for *hif-1αb* [16].

### Statistical Analysis

All data were analysed (Prism 5.0, GraphPad Software) using unpaired, two-tailed t-tests for comparisons between two groups and one-way ANOVA (with Bonferonni post-test adjustment) for other data. P values shown are: *P<.05, **P<.01, and ***P<.001.

## Supporting Information

Figure S1 **Later stage larval infection showed no detectable levels of Hif-1α signaling.** (A) In situ hybridization using a *phd-3* antisense probe indicated no detectable expression in granulomas in 6 dpi zebrafish larvae. Lower panels show larvae treated with DMOG that have upregulated expression of *phd3* indicating that the in situ detection of *phd3* is functional and dependent on activated Hif-α signaling. (B) Micrographs of 4 individual granulomas (encircled with dotted lines) of 6 dpi larvae taken with DIC light microscopy. Hif-α signaling is labeled in the larvae by *phd3* in situ hybridization, however, no levels of expression were detectable in granulomas.
(TIF)

Figure S2 **DMOG did not affect MM bacterial growth** ***in vitro*** **.** (A) Bacterial pixel count of plated out serial dilutions of Mm liquid cultures after overnight incubation in DMSO/DMOG. The bacterial culture was split into two after inoculation and treated with 100 µM DMOG, or DMSO. After treatment and growth overnight, serial dilutions of the liquid culture were plated out and grown for 4 days before imaging. Data shown are mean ± SEM, n = 15 as accumulated from 3 independent experiments. (B) Example fluorescence photomicrographs from the data shown in (A).
(TIF)

Figure S3 **Leukocyte cell-type specific expression of stabilized Hif-1α and macrophage labeling with anti-nitrotyrosine.** (A) Confocal photomicrographs of *Tg(lyz:da-hif-1αb:ires-nlsegfp)* (*lyz:da-hif-1α*) injected mpeg1:mCherry embryos at 2 dpf. IRES-nlsGFP is expressed in cells in the caudal haematopoetic tissue associated with leukocytes and *mpeg1* positive macrophages, but is not present within the same cell. (B) Confocal photomicrographs of *Tg(mpeg1:da-hif-1αb:ires-nlsegfp)* (*mpeg:da-hif-1α*) injected *Tg(mpeg1:mCherryF)ump2* line (*mpeg*:mCherry) at 2 dpf. IRES-nlsGFP is found expressed in the same cells as *mpeg*:mCherry indicating macrophage expression. (C) Confocal micrographs showing anti nitrotyrosine staining in macrophages in the *mpeg*:mCherry at 2 dpf. Upper panels show a nitrotyrosine negative macrophage, which is representative of the majority of the macrophage population. Middle panels show a nitrotyrosine positive macrophage in the absence of infection. Lower panels show a nitrotyrosine positive macrophage in the presence of infection. In both the absence and presence of infection nitrotyrosine positive macrophages are a rare event (approximately <5% of the population).
(TIF)

Figure S4 **Dominant** ***hif-2αa*** **variants exhibit the same effects on** ***phd3*** **expression as the equivalent dominant** ***hif-1αb*** **variants.** (A) Photomicrographs of 24 hpf embryos after injection with dominant active (DA2) and dominant negative (DN2) *hif-*2*αa* constructs or phenol red (PR) as a control, showing expression of the Hif-α target gene *phd3* by in situ hybridization. (B) Fluorescent photomicrographs of 48 hpf *phd3*:GFP embryos injected with dominant active with dominant active (DA2) and dominant negative (DN2) *hif-2αa* constructs or phenol red (PR) as a control. Upper panels show that DA2 increases the expression of phd3:GFP compared to PR and DN2 (white arrows). Lower panels show that DN2 can block the increased expression of *phd3*:GFP in the yolk (white arrows) after DMOG treatment. (C) *phd3*:GFP embryos were injected at the 1 cell stage with dominant negative *hif-2αa* RNA (DN2) or phenol red (PR) as a negative control. 60 embryos of each were screened for *phd3*:GFP expression using confocal microscopy and the 3 brightest areas of *phd3*:GFP expression were imaged and showed co-localization Mm infection. In the DN2 group GFP laser levels and confocal settings were increased until background green fluorescence was visible showing no specific co-localisation with Mm.
(TIF)

Table S1 ***hif-2αa*** **primers used for cloning and site-directed mutagenesis.** Primers used to PCR amplify the zebrafish *HIF-2α* homologue, *hif-2αa* (ZFIN: *epas1a*), and to make the dominant constructs. Dominant active primers are longer as site directed mutagenesis was performed to introduce each mutation individually in separate PCR reactions. PCR products were transformed into the pCR- II-TOPO vector (Invitrogen) and sequence verified. Each *hif-2αa* construct was then inserted into the pCS2+ vector (Invitrogen) from which RNA was transcribed using SP6 enzyme and the mMessage-Machine kit (Ambion).
(DOCX)
