# Supplementary figures and images for: Hypoxia Inducible Factor Signaling Modulates Susceptibility to Mycobacterial Infection via a Nitric Oxide Dependent Mechanism
Source: PLoS Pathog. 2013 Dec 19;9(12):e1003789. doi: 10.1371/journal.ppat.1003789 (PMC3868520; doi:10.1371/journal.ppat.1003789)

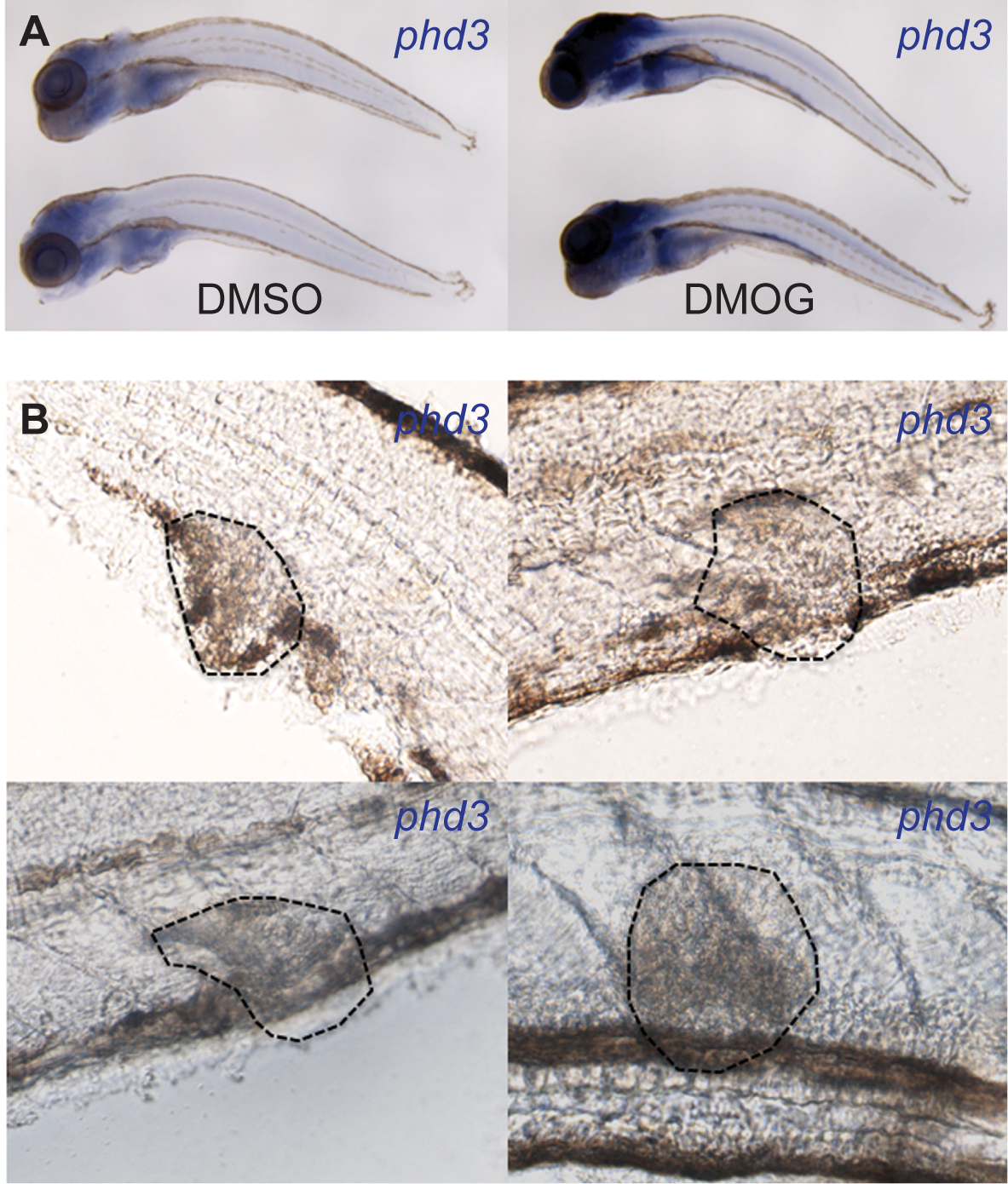

Supplement: Figure S1 — Later stage larval infection showed no detectable levels of Hif-1α signaling. (A) In situ hybridization using a phd-3 antisense probe indicated no detectable expression in granulomas in 6 dpi zebrafish larvae. Lower panels show larvae treated with DMOG that have upregulated expression of phd3 indicating that the in situ detection of phd3 is functional and dependent on activated Hif-α signaling. (B) Micrographs of 4 individual granulomas (encircled with dotted lines) of 6 dpi larvae taken with DIC light microscopy. Hif-α signaling is labeled in the larvae by phd3 in situ hybridization, however, no levels of expression were detectable in granulomas. (TIF) [file ppat.1003789.s001.tif]

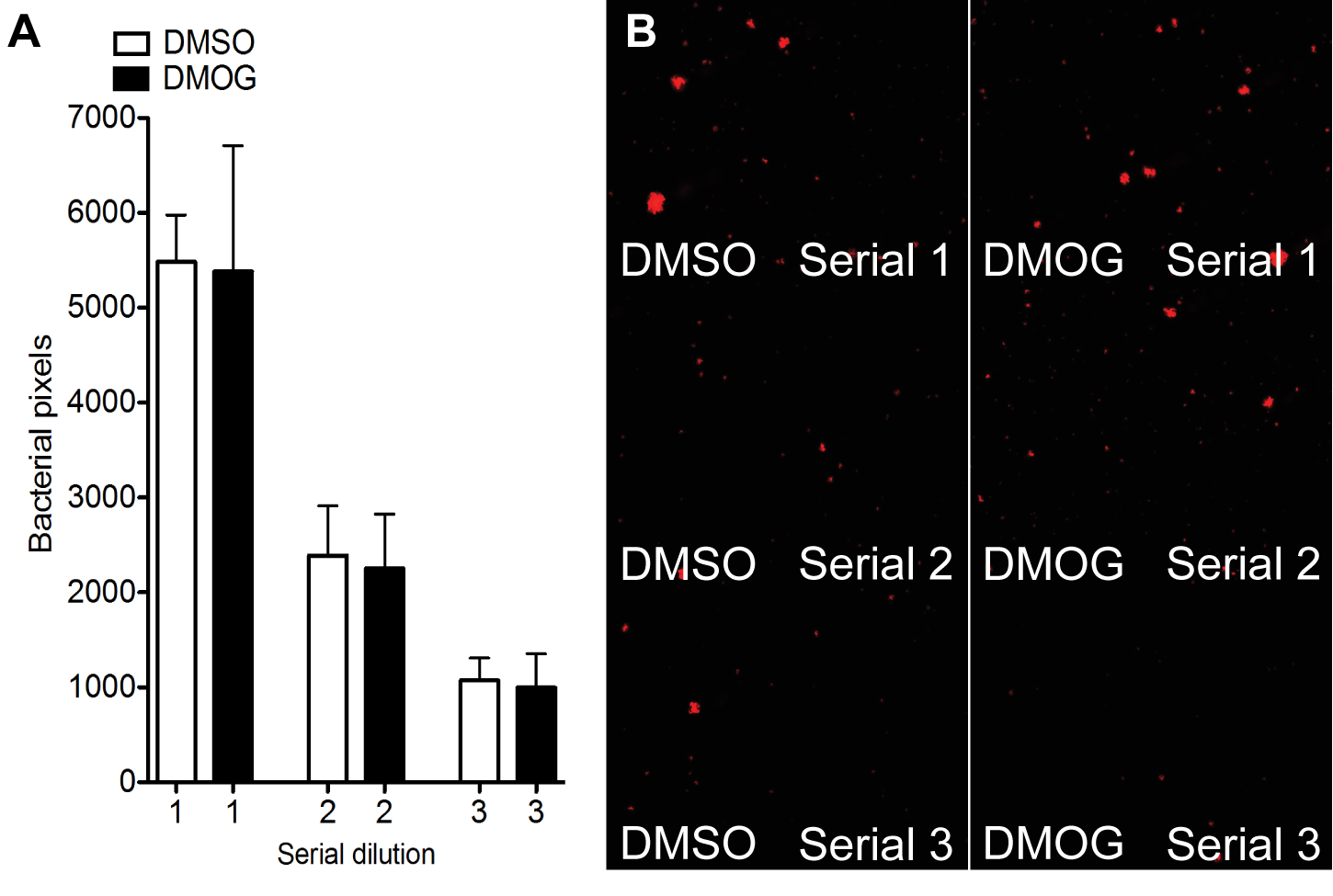

Supplement: Figure S2 — DMOG did not affect MM bacterial growth in vitro . (A) Bacterial pixel count of plated out serial dilutions of Mm liquid cultures after overnight incubation in DMSO/DMOG. The bacterial culture was split into two after inoculation and treated with 100 µM DMOG, or DMSO. After treatment and growth overnight, serial dilutions of the liquid culture were plated out and grown for 4 days before imaging. Data shown are mean ± SEM, n = 15 as accumulated from 3 independent experiments. (B) Example fluorescence photomicrographs from the data shown in (A). (TIF) [file ppat.1003789.s002.tif]

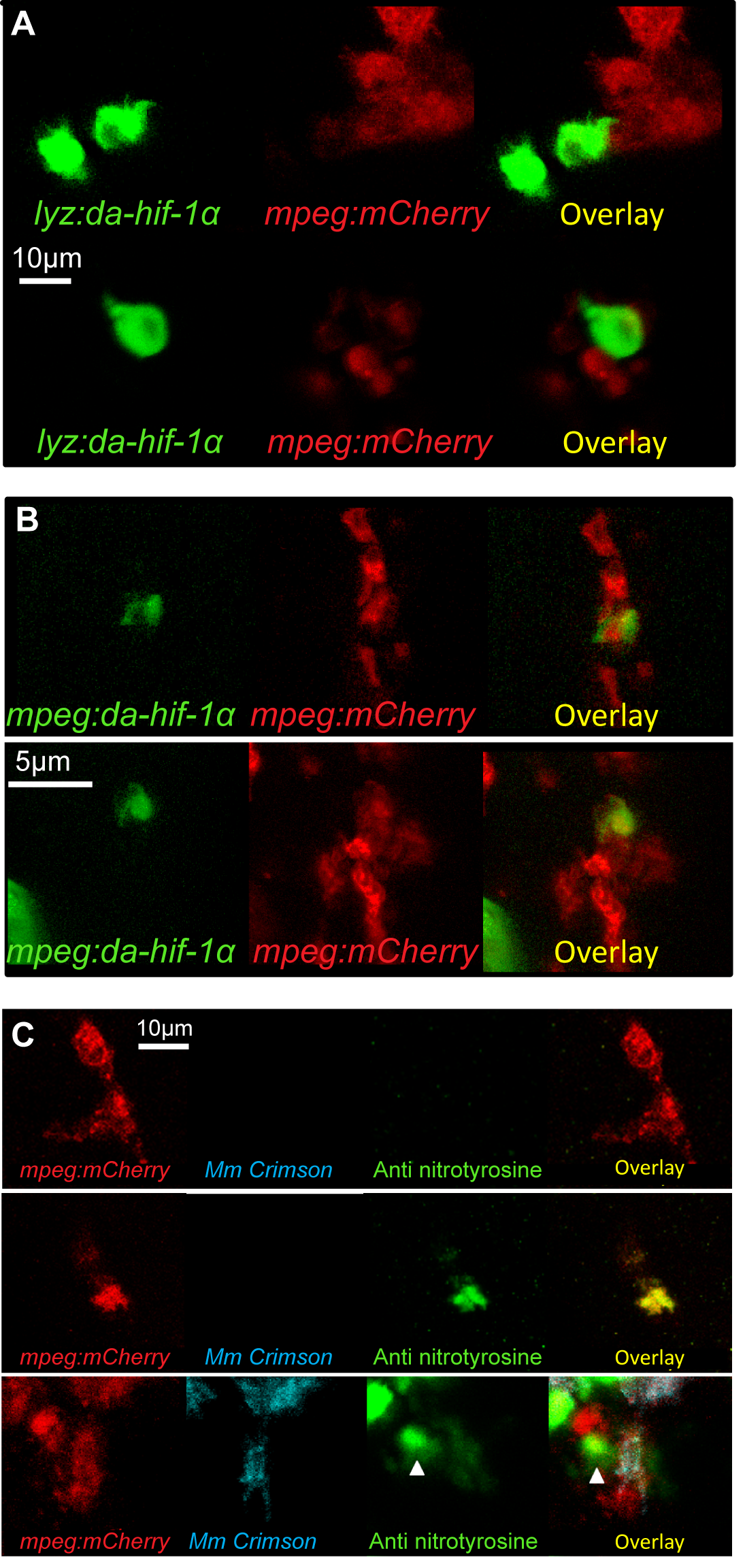

Supplement: Figure S3 — Leukocyte cell-type specific expression of stabilized Hif-1α and macrophage labeling with anti-nitrotyrosine. (A) Confocal photomicrographs of Tg(lyz:da-hif-1αb:ires-nlsegfp) (lyz:da-hif-1α) injected mpeg1:mCherry embryos at 2 dpf. IRES-nlsGFP is expressed in cells in the caudal haematopoetic tissue associated with leukocytes and mpeg1 positive macrophages, but is not present within the same cell. (B) Confocal photomicrographs of Tg(mpeg1:da-hif-1αb:ires-nlsegfp) (mpeg:da-hif-1α) injected Tg(mpeg1:mCherryF)ump2 line (mpeg:mCherry) at 2 dpf. IRES-nlsGFP is found expressed in the same cells as mpeg:mCherry indicating macrophage expression. (C) Confocal micrographs showing anti nitrotyrosine staining in macrophages in the mpeg:mCherry at 2 dpf. Upper panels show a nitrotyrosine negative macrophage, which is representative of the majority of the macrophage population. Middle panels show a nitrotyrosine positive macrophage in the absence of infection. Lower panels show a nitrotyrosine positive macrophage in the presence of infection. In both the absence and presence of infection nitrotyrosine positive macrophages are a rare event (approximately <5% of the population). (TIF) [file ppat.1003789.s003.tif]

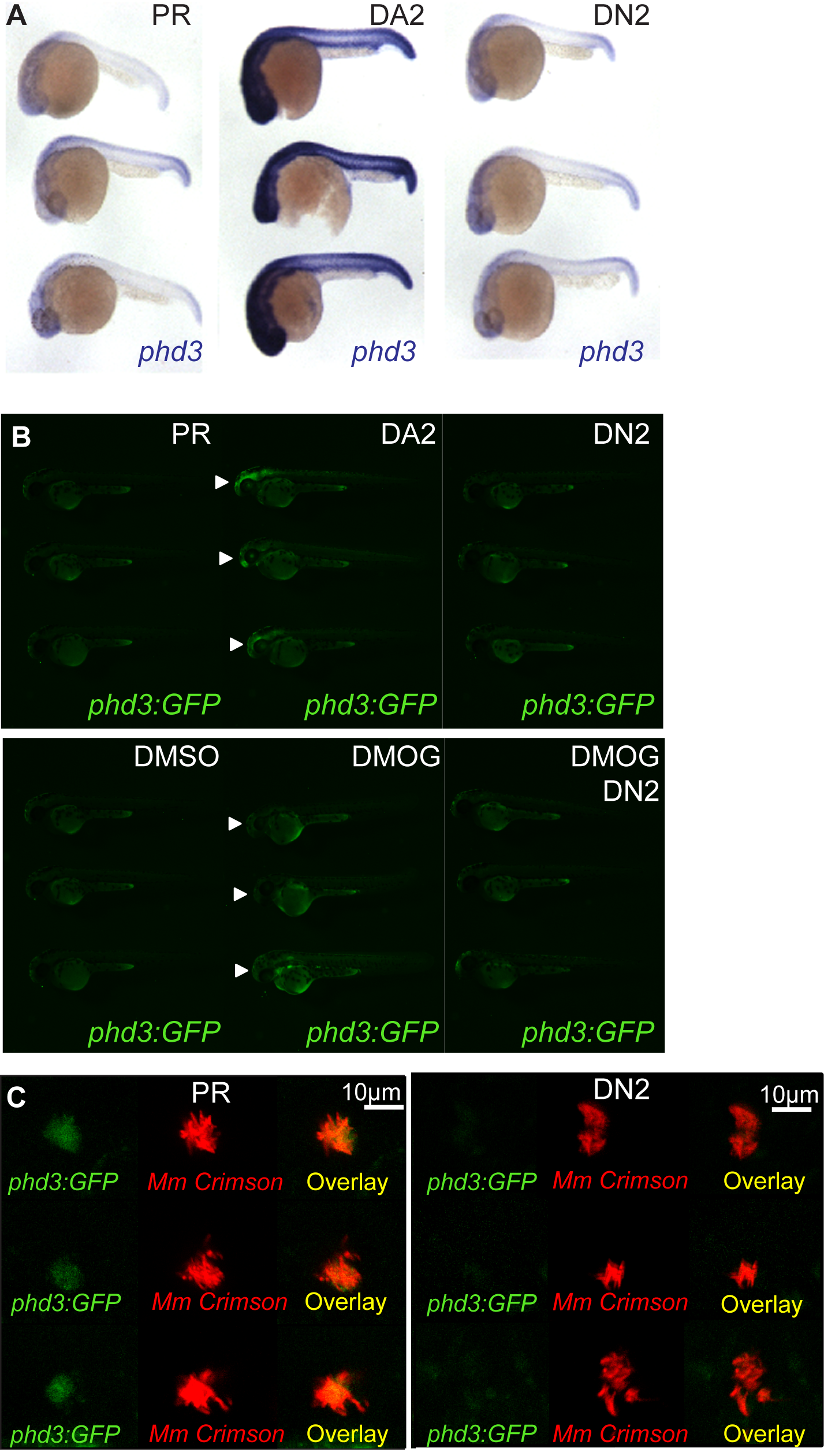

Supplement: Figure S4 — Dominant hif-2αa variants exhibit the same effects on phd3 expression as the equivalent dominant hif-1αb variants. (A) Photomicrographs of 24 hpf embryos after injection with dominant active (DA2) and dominant negative (DN2) hif-2αa constructs or phenol red (PR) as a control, showing expression of the Hif-α target gene phd3 by in situ hybridization. (B) Fluorescent photomicrographs of 48 hpf phd3:GFP embryos injected with dominant active with dominant active (DA2) and dominant negative (DN2) hif-2αa constructs or phenol red (PR) as a control. Upper panels show that DA2 increases the expression of phd3:GFP compared to PR and DN2 (white arrows). Lower panels show that DN2 can block the increased expression of phd3:GFP in the yolk (white arrows) after DMOG treatment. (C) phd3:GFP embryos were injected at the 1 cell stage with dominant negative hif-2αa RNA (DN2) or phenol red (PR) as a negative control. 60 embryos of each were screened for phd3:GFP expression using confocal microscopy and the 3 brightest areas of phd3:GFP expression were imaged and showed co-localization Mm infection. In the DN2 group GFP laser levels and confocal settings were increased until background green fluorescence was visible showing no specific co-localisation with Mm. (TIF) [file ppat.1003789.s004.tif]
