# Supplementary material for: Hypoxia Inducible Factor Signaling Modulates Susceptibility to Mycobacterial Infection via a Nitric Oxide Dependent Mechanism
Source: PLoS Pathog. 2013 Dec 19;9(12):e1003789. doi: 10.1371/journal.ppat.1003789 (PMC3868520; doi:10.1371/journal.ppat.1003789)
Supplement: Table S1 — hif-2αa primers used for cloning and site-directed mutagenesis. Primers used to PCR amplify the zebrafish HIF-2α homologue, hif-2αa (ZFIN: epas1a), and to make the dominant constructs. Dominant active primers are longer as site directed mutagenesis was performed to introduce each mutation individually in separate PCR reactions. PCR products were transformed into the pCR- II-TOPO vector (Invitrogen) and sequence verified. Each hif-2αa construct was then inserted into the pCS2+ vector (Invitrogen) from which RNA was transcribed using SP6 enzyme and the mMessage-Machine kit (Ambion). (DOCX) [file ppat.1003789.s005.docx]

**Supporting Information**

**Hypoxia Inducible Factor Signaling Modulates Susceptibility to Mycobacterial Infection Via a Nitric Oxide Dependent Mechanism**

Philip M. Elks, Sabrina Brizee, Michiel van der Vaart, Sarah R Walmsley, Fredericus J. van Eeden, Stephen A. Renshaw, Annemarie H. Meijer

**Supporting Table**

| **PCR Product** | **Forward Primer** | **Reverse Primer** |
| --- | --- | --- |
| *hif-2αa* | 5’-CACACCTGGACAAAGCC  TCT-3’ | 5’-GGATGAAGAGGGTGAAT  GGA-3’ |
| *dn-hif-2αa*  Δ330 | 5’-CACACCTGGACAAAGCC  TCT-3’ | 5’-GGAGTTGCGGTTGTT  GTA-3’ |
| *da-hif-2αa* P347A | 5’-TAGCGCAGTTAGCGGCTA  TGCCAGGAGAC-3’ | 5’-GTCTCCTGGCATAGCCG  CTAACTGCGCTA-3’ |
| *da-hif-2αa* P481G | 5’-CCTGGAGACTCTCGCTGG  ATACATCCCAATGGAC-3’ | 5’-GTCCATTGGGATGTATC  CAGCGAGAGTCTCCAGG-3’ |
| *da-hif-2αa*  N753A | 5’-GCGATATGACTGTGAGGTAG  CCATGCCTCTACAAGGAAAC-3’ | 5’-GTTTCCTTGTAGAGGCATGG  CTACCTCACAGTCATATCGC-3’ |
